# Conformational Transitions upon Ligand Binding: Holo-Structure Prediction from Apo Conformations

**DOI:** 10.1371/journal.pcbi.1000634

**Published:** 2010-01-08

**Authors:** Daniel Seeliger, Bert L. de Groot

**Affiliations:** Computational Biomolecular Dynamics Group, Max-Planck-Institute for Biophysical Chemistry, Göttingen, Germany; Max-Planck-Institut für Informatik, Germany

## Abstract

Biological function of proteins is frequently associated with the formation of complexes with small-molecule ligands. Experimental structure determination of such complexes at atomic resolution, however, can be time-consuming and costly. Computational methods for structure prediction of protein/ligand complexes, particularly docking, are as yet restricted by their limited consideration of receptor flexibility, rendering them not applicable for predicting protein/ligand complexes if large conformational changes of the receptor upon ligand binding are involved. Accurate receptor models in the ligand-bound state (holo structures), however, are a prerequisite for successful structure-based drug design. Hence, if only an unbound (apo) structure is available distinct from the ligand-bound conformation, structure-based drug design is severely limited. We present a method to predict the structure of protein/ligand complexes based solely on the apo structure, the ligand and the radius of gyration of the holo structure. The method is applied to ten cases in which proteins undergo structural rearrangements of up to 7.1 Å backbone RMSD upon ligand binding. In all cases, receptor models within 1.6 Å backbone RMSD to the target were predicted and close-to-native ligand binding poses were obtained for 8 of 10 cases in the top-ranked complex models. A protocol is presented that is expected to enable structure modeling of protein/ligand complexes and structure-based drug design for cases where crystal structures of ligand-bound conformations are not available.

## Author Summary

Structure-based drug design has become a powerful tool in modern drug discovery pipelines. A critical prerequisite is a structure of the target protein close to its ligand bound conformation which is often difficult to determine experimentally. In many cases, a structure of the unbound receptor is available, but conformational changes with respect to the ligand-bound form preclude it from being used as a basis for structure-based drug design. We have developed a computational approach to predict the structure of protein/ligand complexes based solely on the unbound conformation, the ligand, and easy-to-assess experimental data. We tested our protocol on proteins that undergo substantial structural rearrangements upon binding a ligand and were able to predict structures of protein/ligand complexes which are in good agreement with experimentally determined structures. The ability to predict ligand bound receptor conformations based on structures in the unbound state enables structure-based drug design for cases where crystallization of the complex has not been successful so far.

## Introduction

Interactions between proteins and small molecules are involved in many biochemical phenomena. Insight into these processes relies on detailed knowledge about the structure of protein/ligand complexes, e.g. how enzymes stabilize substrates and cofactors in close proximity. Moreover, almost all drugs are small-molecule ligands that interact with enzymes, receptors or channels. Accordingly, ligand-bound receptor complex structures are a critical prerequisite for understanding biological function and for structure based drug design. However, structure determination of protein/ligand-complexes can be difficult, time-consuming and expensive. Crystal structures of protein/ligand complexes are usually obtained either by co-crystallization or soaking and it is a common problem that even when conditions for crystallizing the apo-protein are well established these might not be transferable to the protein/ligand complex [1]–[4]. Particularly, conformational transitions of the receptor associated with ligand binding pose a severe challenge to the structure elucidation of holo complexes [5]–[8].

When structures of ligand-bound protein conformations are not available, structure-based drug design becomes highly challenging. Several studies showed that virtual screening to an apo-structure usually results in a poor enrichment factor (the ability to discriminate between binders and non-binders) compared to the holo-structure even when the structural difference between both is comparably small [9]–[11]. Therefore, the development of docking programs aims at allowing a certain degree of receptor flexibility either by using an ensemble of structures instead of a single receptor conformation [12]–[15] or by explicitely modeling flexibility such as sidechain variations (Autodock4 [16],[17], Gold [18],[19], FlexX [20], RosettaLigand [21]), predefined flexibility of certain parts of the structure (FlipDock [22]) and also small variations of the backbone (Glide/Prime [23], RosettaLigand [24], ICM [25],[26]). Incorporating receptor flexibility in molecular docking is a substantial progress and has been shown to enhance both enrichment factors and the ability to predict correct binding poses, particularly in cases when docking a compound to a receptor structure that has been crystallized with a different ligand (cross-docking) which is usually the case when searching for novel drugs. However, the degree of flexibility thus far is limited to either sidechain motions or small variations of the backbone and thus, the availability of a holo-structure or an apo-structure that is highly similar to the holo conformation is currently a prerequisite for a successful docking, severely limiting structure-based drug design.

Particularly, receptors that undergo a substantial conformational transition upon ligand binding are currently precluded from structure based drug design.

Although protein-ligand crystals suitable for diffraction might not be accessible, several experimental techniques exist to detect conformational changes. In many cases where proteins undergo domain reorientations upon ligand binding they adopt a different shape in the ligand bound state, corresponding to a change in the radius of gyration that can be studied either by NMR, where a more compact shape causes a descrease in the rotational correlation time [27]–[29] or by small-angle scattering of x-rays (SAXS) or neutrons (SANS) [30]–[33]. These shape descriptions provide invaluable information for modeling of structures [34]–[36] and macromolecular assemblies [37],[38] as well as insight into protein dynamics.

**Figure 1:**
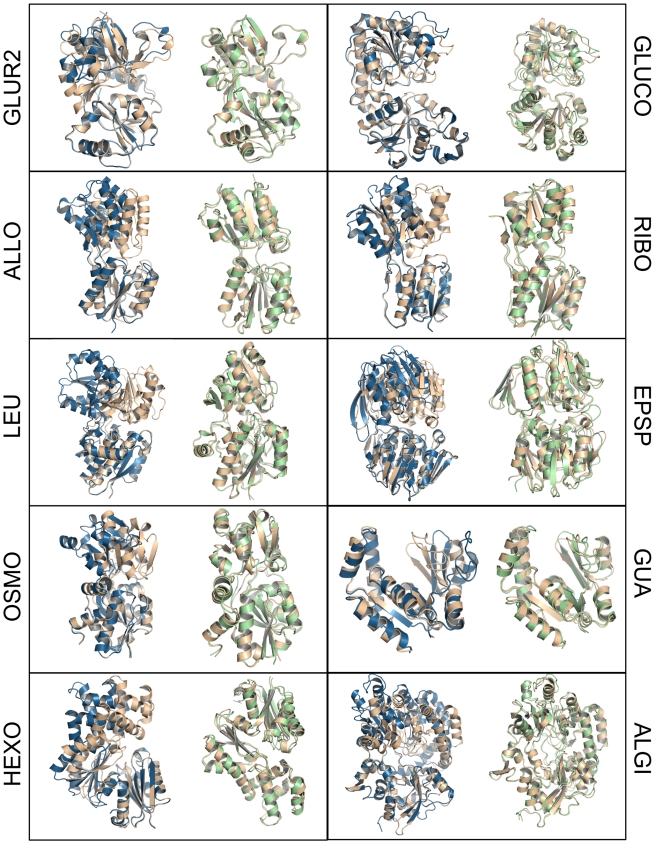
Overlay of apo/holo pairs and holo/model pairs. Receptor models were predicted based on the apo-structure, the ligand and the radius of gyration of the holo conformation. Apo x-ray structures are shown in blue, holo x-ray structures are colored wheat. The best modelled receptor structure is shown in green.

Here, we present a method to predict the structure of protein/ligand complexes for proteins that undergo a large conformational change upon ligand binding. The protocol solely requires the apo-structure, a known ligand and experimental data on the shape of the holo-structure. Here, we apply the radius of gyration as shape information, a quantity that can frequently be readily assessed more easily than an x-ray structure. We developed a simulation protocol that combines biased conformational sampling, docking and molecular dynamics simulations and applied it to ten ligand-binding proteins (see Table 1). We chose cases where both, the unbound conformation and the bound conformation are known from x-ray crystallography in order to be able to a-posteriori validate the predicted receptor conformations and docking poses. The conformational changes involved range from 2.1 to 7.1 Å backbone RMSD (see table 1 and fig. 1) and the binding site geometries differ substantially between the apo and the holo conformations. In nine of the ten cases we predict holo receptor conformations close to the native ligand-bound conformation and in eight cases we predict ligand binding poses close to the native state, rendering our method suitable for blind predictions of protein/ligand complexes involving large conformational transitions.[Table pcbi-1000634-t002]

**Table 1:** Comparison of apo/holo pairs of proteins.

Receptor	Abbreviation	PDB*_apo_*	PDB*_holo_*	# Res.	Ligand	RMSD*_BB_*	RMSD*_BS_*
GluR2 ligand binding core	GLUR2	1fto	1ftm	257	AMPA	2.2	2.0
DNA Beta-Glucosyl-transferase	GLUCO	1jej	1jg6	351	Uridine-5′-diphosphate	2.1	2.6
D-Allose binding protein	ALLO	1gud	1rpj	288	D-Allose	4.4	4.0
D-Ribose binding protein	RIB	1urp	2dri	271	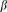 -D-Ribose	4.3	3.5
L-Leucine binding protein	LEUB	1usg	1usi	345	Phenylalanine	7.1	6.8
5-Enolpyruvylshikimate-3-phosphate synthase	EPSP	1rf5	1rf4	427	SPQ	3.7	4.6
Osmo-protection protein	OSMO	1sw5	1sw2	270	Glycine-betaine	5.0	4.4
Guanylate kinase	GUA	1ex6	1ex7	186	GMP	3.6	3.9
Hexokinase	HEXO	2e2n	2e2o	298	Glucose	3.0	1.9
Alginate binding protein	ALGI	1y3q	1y3n	490	Alginate Disaccharide	4.8	3.6

RMSD*_BB_*: Root mean square deviation of all backbone atoms after least square fit. RMSD*_BS_*: Root mean square deviation of the binding site. The binding site was defined as all residues which have at least one atom within 6 Å of any ligand atom.

## Results

The proteins used in this study undergo large conformational changes upon ligand binding. The conformational changes involve large domain reorientations, accompanied by loop motions and sidechain movements. Since we use the radius of gyration as an example of an experimentally determined constraint we chose cases where a distinct change of this observable takes place upon ligand binding. This is usually the case for protein motions which are classified as hinge-bending motions according to the database of macromolecular movements [6]. However, such a classification is not necessary for the described protocol and is not used for the predictions made in this work. Here, we focus at cases that undergo domain closure upon ligand binding. With that aim, constraints which enforce domain closure were incorporated into the tCONCOORD program, thereby enhancing the sampling around the closed state significantly (see Text S1).

The ten proteins used for this study belong to five different SCOP [39]–[41] superfamilies.

- Periplasmic-binding protein type I: ALLO, LEU, RIBO
- Periplasmic-binding protein type II: OSMO, ALGI, GLUR2
- EPT/RTPC-like: EPSP
- P-loop containing nucleoside triphosphate hydrolase: GUA
- Actin-like ATPase domain: HEXO, GLUCO

Periplasmic-binding proteins (PBPs) mediate a wide range of fundamental processes and are ubiquitous in bacteria. Therefore, they are discussed as potential targets for antmicrobial agents. Moreover, the general “Venus-flytrap”- architecture of PBPs is also found in the extracellular ligand-binding domain of class C G-protein coupled receptors (GPCRs) many of which are of great interest as drug targets [42]. The other cases belong to different classes of kinases which represents another large group of pharmacologically interesting receptors.

Table 1 lists the protein data bank [43] (PDB) codes of the apo/holo pairs, their respective ligands and the global backbone root mean square deviations (RMSD) as well as the RMSDs of the binding site.

In all cases, the ligand binding pocket differs substantially between the bound and the unbound state or is virtually not present in the apo conformation. A structure prediction protocol therefore needs to precisely predict the domain motions as well as local structural rearrangments. We use the tCONCOORD program [44] to generate an initial ensemble of conformations which share a predefined radius of gyration. Since the constraints tCONCOORD uses for the structure prediction are derived from a given protein structure (here the apo conformation) the resulting ensemble represents an approximation of the conformational space that is accessible within the predefined constraints. Hence, if interactions like hydrogen bonds are defined as constraints in the input structure they will be conserved in the resulting ensemble. If conformational changes are associated with partial unfolding or refolding, resulting in major changes of, e.g. the hydrogen bond network, the generated ensemble will be likely to miss those conformations. Domain motions however, are ususally well predicted [44]. The first part of the structure prediction protocol therefore aims at predicting the correct orientation of domains. The molecular dynamics simulations carried out subsequently basically serve as a filter to distinguish between correct models and those with correct geometry but unfavourable energetics which tend to re-open quickly.

### 

#### Prediction of holo receptor structures

**Figure 2:**
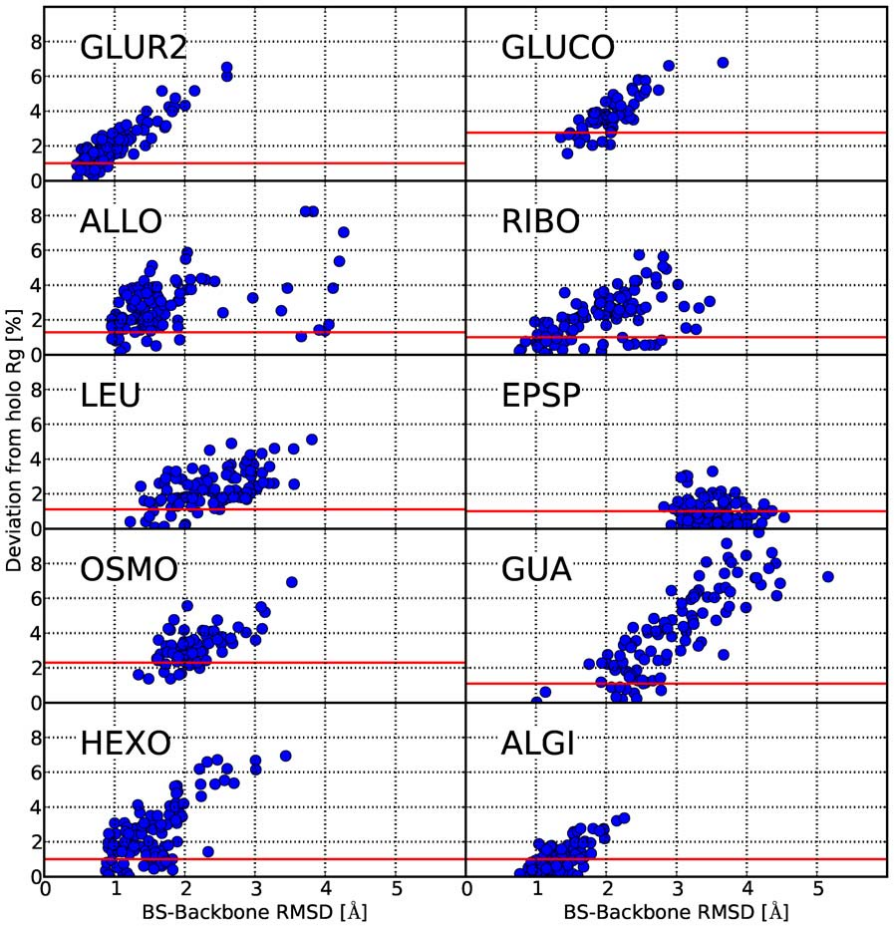
Binding-site RMSD vs. deviation from holo Rg. The deviation from the target radius of gyration correlates well with low RMSD's to the experimentally determined holo structures. The best receptor models (indicated by the red line) were used for binding site refinenment with Rosetta.

The results are summarized in Table 2. After the refinement cycles we find a good correlation between the radius of gyration and the RMSD to the holo structures (see fig 2). In all of the 10 cases the global backbone RMSD of the best model to the known holo structure is below 1.6 Å (fig. 1). For the prediction of ligand binding poses, however, it is more important to focus on the geometry of the binding site. Here, we find that the prediction of the arrangement of the backbone atoms belonging to the binding site is equal or below 1 Å RMSD accuracy in six of the ten cases and below 1.4 Å in nine cases. An RMSD calculated over all atoms (including the sidechains) corresponding to the binding site reveals RMSD values below 2 Å for nine of the ten cases. EPSP, where the backbone deviation is 2.8 Å represents a case were the current protocol reaches its limitations since here, the structural rearrangment between apo and holo structure involves refolding of a helix into a loop, a transition which is not predicted by the current sampling protocol. This causes some residues in the predicted structures to be more than 10 Å away from their target destination, hence resulting in a large RMSD.

**Table 2 pcbi-1000634-t002:** Model accuracy.

Receptor	Overall BB-RMSD	BS BB-RMSD	BS all-atom RMSD	Rank 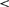 1.5 Å BB
GLUR2	0.91	0.46	0.84	1
GLUCO	1.38	1.35	1.78	10
ALLO	1.07	0.96	1.55	2
RIBO	0.98	0.76	1.2	3
LEU	1.15	1.2	1.9	5
EPSP	1.58	2.8	3.58	-
OSMO	1.27	1.3	1.96	2
GUA	1.45	1.0	1.76	2
HEXO	1.42	0.87	1.38	3
ALGI	1.27	0.77	1.5	3

The table shows RMSD values for the best models. In all cases the global backbone RMSD is below 1.6 Å. The backbone RMSD of the binding site is below 1 Å in 5 cases and the the RMSD calculated over all heavy atoms in the binding site is below 2 Å in 9 of 10 cases. Rank 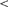
1.5 Å BB gives the rank of the first model to get within 1.5 Å RMSD to the holo x-ray structure.

#### Prediction of ligand binding poses

**Figure 3:**
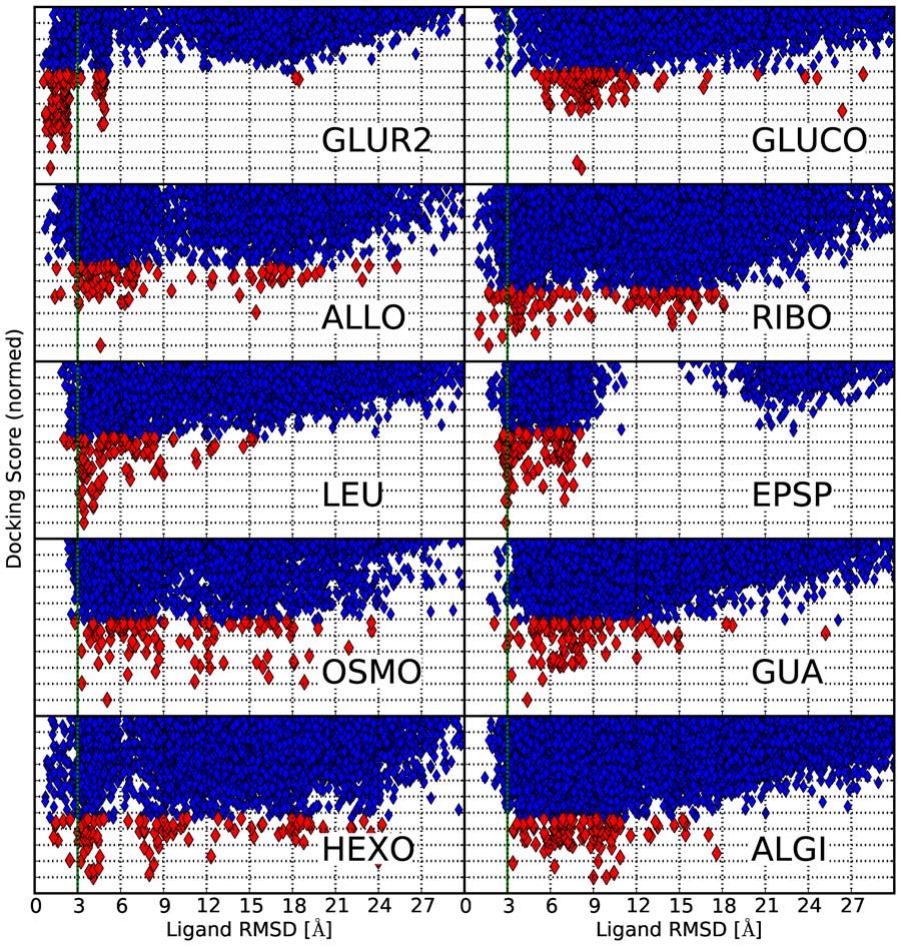
Ligand RMSD's vs. Rosetta Score. The plot shows the RMSD's of ligand poses generated with RosettaLigand. In all cases ligand poses below 3 Å to the target structure have been sampled and in 8 cases a pose below 3 Å RMSD is found within the top 100 (red) of the ranked poses.

**Figure 4:**
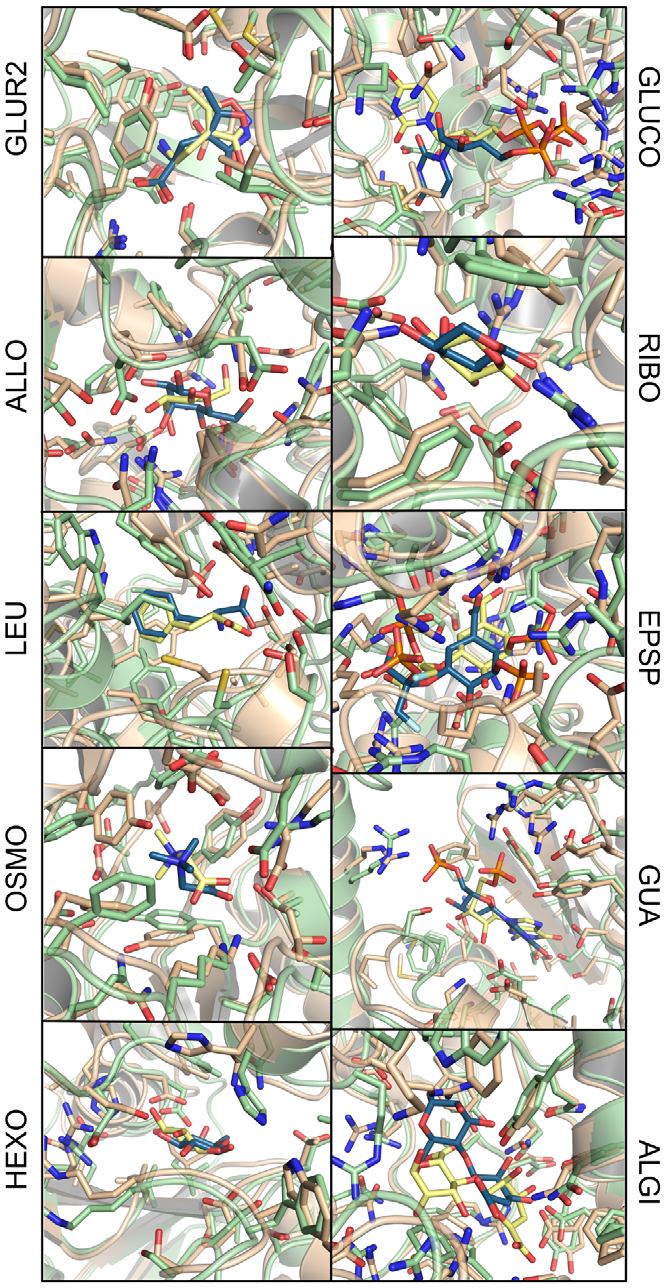
Modeled protein/ligand complexes. X-ray structures of receptor and ligand are shown in wheat and yellow, modeled complexes are colored green/blue.

Obtaining close-to-native ligand binding poses with receptor structures obtained from structure modeling or low-resolution data is a non-trivial task even when close-to-native receptor conformations are available. Scoring functions in docking programs are usually very sensitive to even small conformational variations or structural inaccuracies and a single misplaced sidechain can preclude successful docking. Therefore, low binding site RMSD's do not automatically translate into low ligand RMSD's. Fig. 3 shows a plot of the ligand RMSD versus the RosettaLigand energy. As can be seen, ligand binding poses close to the native state were sampled in all cases but, due to structural variations in the binding site, these were not always among the top ranked configurations. In 8 of the 10 cases, however, ligand binding poses below 3 Å RMSD to the X-ray structure are found within the top ranked solutions (see fig. 4). In docking studies to rigid receptors ligand RMSDs of 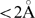 to the experimentally determined binding pose are usually regarded as successful. In our case the ligand RMSD was calculated after least square fitting of the modeled complex to the target structure. Hence, deviations of both, the modeled receptor structure and the ligand pose contribute to the calculated ligand RMSDs.

## Discussion

In this work, we present a proof of concept study that allows successful prediction of holo-receptor conformations and ligand binding poses. With an approach based solely on the receptor in its unbound conformation, a known binder, such as the natural substrate, and relatively easy to assess shape information on the ligand-bound state, we predicted receptor models within 1.6 Å RMSD to the x-ray holo structure starting from apo conformations that deviate up to 7.1 Å RMSD from their respective ligand-bound state. For 8 of the 10 cases ligand binding poses within 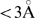 RMSD to the x-ray structure were predicted, underpinning the applicability of our method to predict receptor models suitable for virtual screening.

With the described protocol we bridge a gap arising from the difficulties associated with structure determination of protein/ligand complexes, the limited conformational flexibility that can be incorporated in docking programs and the limited sampling that can be achieved with molecular dynamics simulations. If apo and holo conformations of a protein differ substantially, current computational structure prediction approaches are usually unable to provide accurate receptor models. As docking protocols need to be fast to be suitable for virtual high-troughput screening they always represent a trade-off between speed and accuracy. Incorporation of sidechain conformational flexibility and backbone plasticity is a substantial progress but each additional degree of freedom is accompanied with growing computational demand and, hence, allowing for large conformational changes has thus for not been feasible. Molecular dynamics simulations are in principle capable of sampling holo conformations when starting from an apo protein. But, in addition to the general sampling problem MD simulations suffer from, ligands may cause alterations in the free energy landscape. Depending on the differences in the free energy landscapes of the apo protein and the protein bound to a ligand, the sampling of close-to-holo receptor conformations without a ligand present may be particularly poor.

The protocol described in this work combines the strengths of docking, conformational sampling and molecular dynamics while overcoming their particular limitations. In contrast to previous studies [45]–[48] it is neither restricted to a particular type of protein motion nor does it require the definition of rigid parts or hinges in a structure or any other approximation. Protein flexibility is accounted for on each level from domain motions to sidechain fluctuations and receptors and ligands are treated as all atom or heavy atom models in each state of the protocol. As the sampling of large conformational transitions and crossing of potential energy barriers is handled by the efficient concoord [49] algorithm and MD simulations are used in refinement we gain both efficient sampling and high accuracy within the limitations of the force field.

The protocol furthermore allows incorporation of various experimental data. In this work we used the radius of gyration of the holo conformation but in principle any kind of data can be used that is transferable into geometrical constraints, e.g. data from FRET experiments or mutagenesis studies. Each additional piece of information confines the sampled conformational space and, hence enhances sampling of conformations around a conformational state of interest (see also Text S1). Additional improvement may be achieved by applying filters to the docked configurations. For example, in the initial docking screens all poses were taken into account without further screening. Analysis of the generated poses and discarding e.g. those that are attached to the surface rather than docked into the binding site would further contribute to the enrichment of promising models. As in all structure prediction protocols, sampling is the limiting factor. In most cases, docking of the ligand to the holo x-ray structure yields a lower score than in the modelled receptor structures. Hence, the docking score is usually sufficient to discriminate between the native structure and a receptor model and if the *true* conformation is sampled it is most likely detectable via the docking score. However, despite the relative simplicity to distinguish between the true structure and a model it is very difficult to distinguish between a model with 1 Å RMSD and a model with 5 Å RMSD to the native state. When applying the current protocol to a blind prediction it would therefore be beneficial to incorporate as much experimental data as possible to reduce the conformational space that needs to be explored and to enhance sampling around the *true* ligand-bound conformation. The problem of conformational sampling furthermore increases with the conformational flexibility of the ligand. Although the ligands discussed in this work are less complex than typical drugs they depict the necessity of very accurate binding sites for a successful docking. Sugar ligands and their derivatives, which some of our ligands belong to, contain many hydroxyl groups and are almost symmetric. Thus, rotating the molecule by 180 degrees ofters almost the same scope of interaction to the receptor but the resulting RMSD would not characterize such a pose as a successful docking. Water molecules can also play an important role in ligand binding. In the present study we removed all crystallographic water molecules from the input structures since it is quite challenging to judge which water molecules might also be present in the ligand bound state, which are replaced by parts of the ligand or which might even be essential for ligand binding.

### 

#### Concluding remarks

Overall, we find that our protocol is successful in predicting close-to-native receptor models and, with limitations, close-to-native ligand poses. Starting from apo structures displaying global backbone RMSDs up to 7.1 Å we were able to predict holo structures within 1.6 Å for all of the ten cases and ligand poses within 3 Å RMSD for 8 of the 10 proteins. It offers a robust protocol for accurate structure prediction of protein/ligand complexes and allows structure based drug design for proteins that undergo large conformational changes upon ligand binding, for which only an apo-structure and a shape description of the holo-structure is available.

## Methods

**Figure 5:**
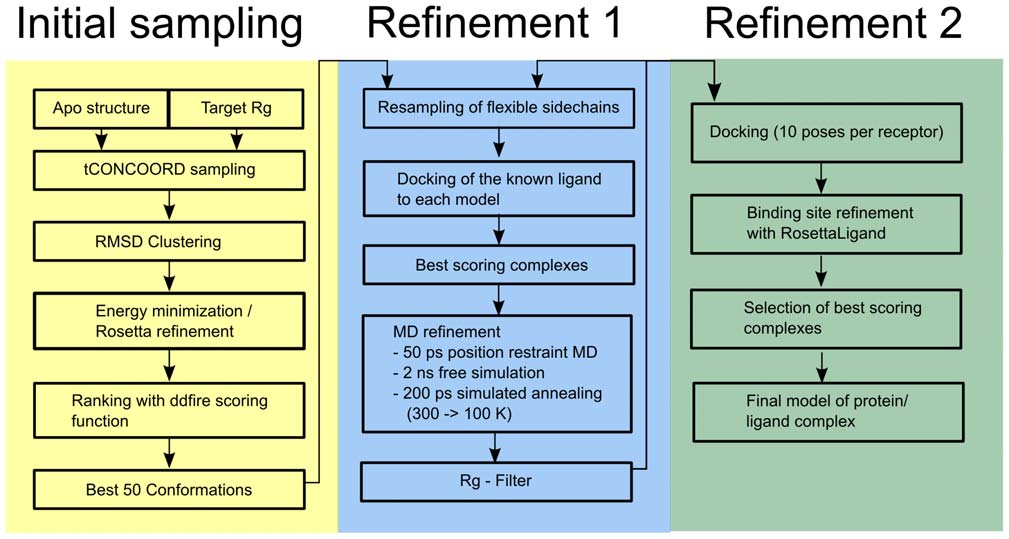
Flowchart of the modeling process. The procedure starts with a biased tCONCOORD sampling that takes the apo conformation and the radius of gyration of the target structure as input. An ensemble of 1000 conformations is generated and, after energy minimization, RMSD clustering and Rosetta refinement, ranked according to the ddfire scoring function. The best 50 conformations were subjected to a tCONCOORD-based sidechain resampling protocol that generates 100 conformations from each input structure with fixed backbone. Afterwards the ligand is docked into each of the 5000 conformations and the 50 best scoring solutions were subjected to molecular dynamics refinement protocol, consisting of 50 ps position restrained MD, followed by 2 ns free simulation and 200 ps simulated annealing. From those models that remained in a closed conformation (close to the holo radius of gyration) another ensemble was generated and subjected to a second refinement cycle. The best models were taken and initial docking poses generated with VINA and refined using the RosettaLigand protocol. The final ranking was carried according to the RosettaLigand score.

The workflow of the presented protocol combines biased conformational sampling, docking and molecular dynamics simulations (see fig. 5).

### 

#### Biased conformational sampling

In a first step, a conformational ensemble is generated based on the apo-structure and the radius of gyration of the target. In this study we did not use experimentally determined radii of gyration but calculated them from the ligand-bound conformations according to
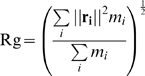
where 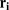 denotes the position of atom *i* with respect to the center of mass of the protein and 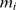 the respective mass. When using experimentally determined values of the radius of gyration one should be aware of the fact that these usually closely match calculated values but are systematically larger due to the solvation shell of the protein [50]. Larger deviations may be a hint towards oligomerization in solution.

The conformational ensembles were generated using tCONCOORD [44],[51]. In previous studies [44],[51] we showed that tCONCOORD is able to generate structure ensembles of proteins from a single input structure, representing a reasonable approximation to the functionally relevant conformational space. The method rests on a thorough analysis of the input structure and the translation of conserved interactions like topological restrictions (bonds, angles), hydrogen bonds and hydrophobic clusters into geometrical constraints with upper and lower bounds. Crystallographic water molecules were removed from the input structures since these are often replaced by the ligand in the bound state. In a second step, these constraints are used to rebuild the structure starting from random atomic coordinates yielding a new structure that fulfills all predefined constraints but differs from the input structure, hence representing an alternative conformation. Typically, an ensemble of 100–10000 independent structures is generated. When generating such an ensemble starting from an apo-protein structure conformations close to the holo receptor conformation are usually included in the ensemble.

If structural information of the ligand-bound state is available from experimental data, it can be used as constraint to bias the ensemble to a conformational subspace of interest [51]. A novel implementation in tCONCOORD allows to additionally use the radius of gyration as geometrical constraint. Thus, with a tCONCOORD simulation using the apo-structure as input, the radius of gyration of the ligand-bound state can be used to enhance the probability of sampling of a conformational subspace of interest, here the ligand-bound state. A radius of gyration bias, however, does not represent such a strong bias as, e.g a distance constraint which enforces a domain closure. Therefore, the ensemble properties are relatively robust against variations of the target radius of gyration and the tolerance used for sampling (see Text S1 for details about biased tCONCOORD samplings).

Initial ensembles of 1000 conformations were generated with tCONCOORD using heavy-atom representations of the receptor structure. Constraints were defined for bonds, angles, 1–4 pairs, hydrogen bonds that meet the desolvation stability criteria [44] and hydrophobic clusters. The radius of gyration of the target structure was used as additional constraint with a tolerance of 0.1 Å to enhance sampling around the ligand-bound conformation. The resulting ensemble was subjected to an RMSD-clustering to reduce the dataset to 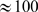 conformations and each conformation was subsequently refined using a 250 steps steepest descent energy minimization in vacuum and 500 steps conjugate gradient minimization in explicit solvent. Afterwards the structures were subjected to a Rosetta refinement and scored with the ddfire [52] scoring function. The best scoring 50 structures were selected for resampling. The tCONCOORD resampling protocol employs random perturbations on sidechain rotamers which are predicted to be flexible based on interaction and packing considerations while keeping the rest of the structure fixed. From each model, 100 conformations where generated, yielding a total number of 5000 protein conformations.

#### Docking and refinement

Afterwards, the known ligand was docked into each of the 5000 models using Autodock VINA [53]. Receptor and ligand preparations were carried out using the AutoDockTools [16]. The binding site was defined as a cubic box of 26.25 Å length placed at the center of the reference ligand position. (Hence, for blind predictions a rough knowledge about the location of the binding site would be required). In all cases the docking box was large enough to guarantee independence of the docking results from small variations of the binding site definitions. From the docking solutions, the best 50 scoring protein/ligand complexes were selected and subjected to 50 ps molecular dynamics simulation with position restraints, followed by 2 ns free simulation and 200 ps simulated annealing to 100 K.The MD simulations were carried out with the Gromacs-4.0 [54] molecular dynamics package in explicit solvent using the Amber03 force field [55],[56] and the tip3p water model [57]. The systems were solvated and NaCl was added to achieve a 150 mM concentration. Ligand parameters were obtained with the generalized Amber force field (GAFF) approach [58]. Electrostatic interactions were calculated at every step with the particle-mesh Ewald method [59], short-range repulsive and attractive dispersion interactions were simultaneously described by a Lennard-Jones potential, which was cut off at 1.0 nm. The SETTLE [60] algorithm was used to constrain bonds and angles of water molecules, and LINCS [61] was used for all other bonds, allowing a time step of 2 fs. The temperature was kept constant at 300 K by weakly coupling the system to an external heat bath [62],[63] (time constant 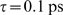) and the pressure was kept constant at 1 atm by weak isotropic coupling to a pressure bath (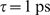).

The resulting structures were ranked by their compliance with the target radius of gyration and the best 10 models were subjected to a second refinement cycle consisting of resampling, docking and, after filtering the best poses, to molecular dynamics refinement.

#### Model assessment and binding site refinement

From the resulting receptor structures the radius of gyration was calculated and those models that had less than 1% deviation from the holo radius of gyrations were chosen for a binding site refinement (In two cases we chose a larger Rg-cutoff since no models were obtained within 1%). For the refinment we carried out docking to the rigid receptor structures and used RosettaLigand to refine the docking pose while simultaneously repacking the sidechains around the ligand. 100 conformations were generated based on each ligand pose and the resulting complexes were ranked according to the RosettaLigand score.

The current protocol requires about four days per case on a 50 node cluster with the most time spent in the molecular dynamics part and the QM based ligand parametrization with gaussian [58],[64].

## Supporting Information

Figure S1 Comparison of free and biased tCONCOORD samplings of D-Ribose binding protein.
(1.39 MB TIF)

Text S1 Comparison of different biased and unbiased sampling protocols in tCONCOORD.
(0.02 MB PDF)
