# Supplementary figures and images for: Conformational Transitions upon Ligand Binding: Holo-Structure Prediction from Apo Conformations
Source: PLoS Comput Biol. 2010 Jan 8;6(1):e1000634. doi: 10.1371/journal.pcbi.1000634 (PMC2796265; doi:10.1371/journal.pcbi.1000634)

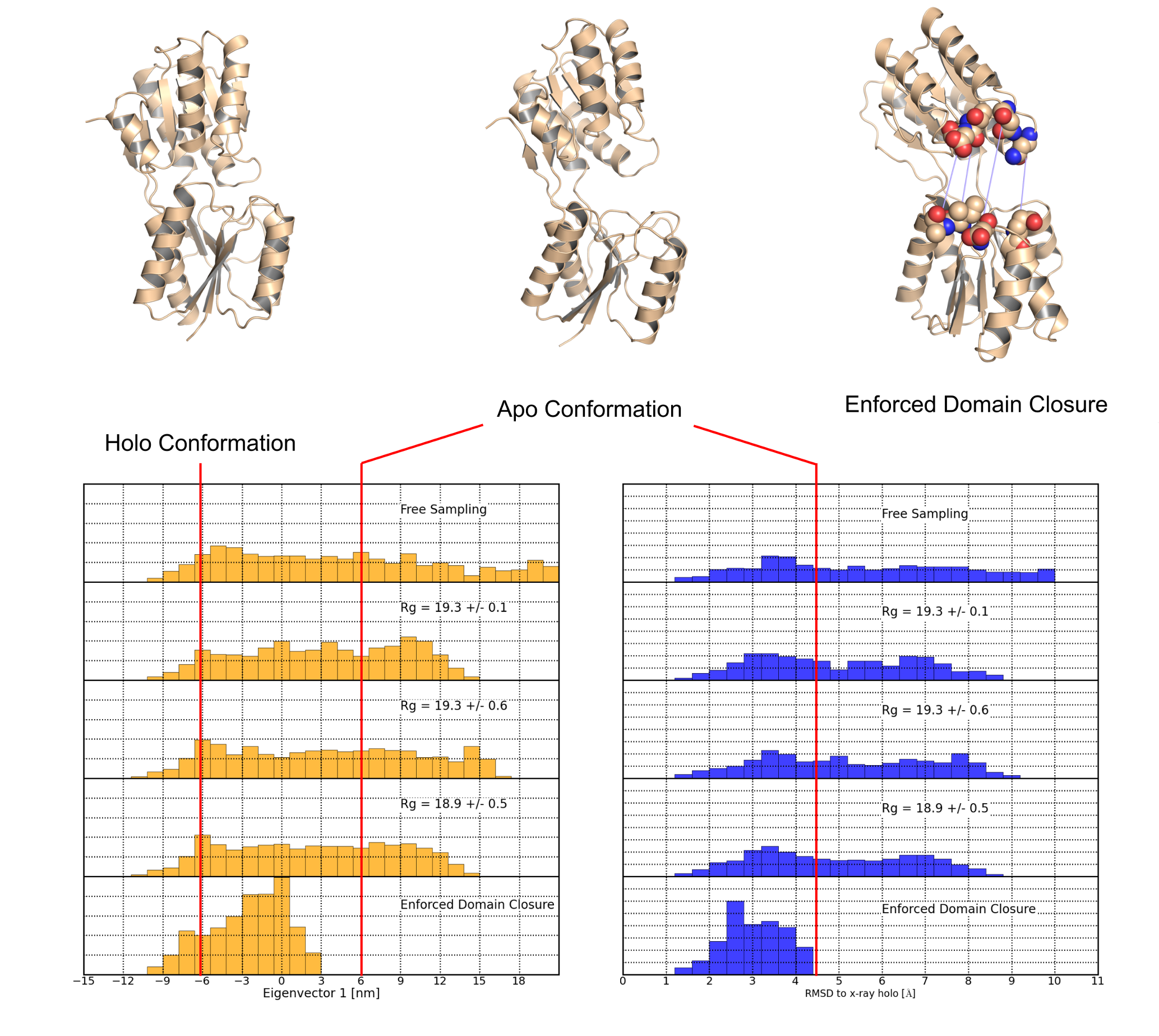

Supplement: Figure S1 — Comparison of free and biased tCONCOORD samplings of D-Ribose binding protein. (1.39 MB TIF) [file pcbi.1000634.s001.tif]
