# Supplementary material for: Conformational Transitions upon Ligand Binding: Holo-Structure Prediction from Apo Conformations
Source: PLoS Comput Biol. 2010 Jan 8;6(1):e1000634. doi: 10.1371/journal.pcbi.1000634 (PMC2796265; doi:10.1371/journal.pcbi.1000634)
Supplement: Text S1 — Comparison of different biased and unbiased sampling protocols in tCONCOORD. (0.02 MB PDF) [file pcbi.1000634.s002.pdf]

Text S1

Figure S1 shows a comparison of tCONCOORD samplings of D-Ribose binding protein with different biasing schemes. We performed an unbiased sampling, one run with a radius of gyration (Rg) constraint around the Rg of the closed state (19.3 Å) with a tolerance of +/- 0.1 Å as used in the paper, one run with the same target Rg but a larger tolerance of +/-0.6 Å and one run with a smaller target Rg of 18.9 Å and a tolerance of +/-0.5 Å . For comparison we also generated an ensemble were we employed distance constraints between residues of the two domains to enforce the generation of more closed conformations. The histograms on the left show the distribution of generated structures along the PCA (principal component analysis) eigenvector that describes the transition from the open to the closed state. As can be seen the influence of a Rg bias on the generated ensembles is limited. Only conformations which correspond to more open states than the apo conformation (right side of the histgrams) are excluded through the Rg bias but the diversity of the ensembles is still large. A larger tolerance or a smaller target Rg also don't affect the distribution of the structures in the conformational space significantly. This behaviour is also observed if the enrichment of structures around the closed state is regarded. The histograms on the right where the distribution of the ensemble with respect to the RMSD to the closed state is shown reveals that a Rg bias does not necessarily produce an enrichment of structures close the ligand bound conformation, although we found that this depends on the particular case. Only structures far away from the target are excluded from the Rg-biased ensembles. The last panel in both graphs shows the ensmble generated with distance constraints to enforce domain closure. Here we observe a strong bias towards the closed state and a much smaller conformational diversity of the ensemble. Hence, if there are experimental hints that a change in the Rg is caused by a closure motion, a direct enforcement of the closing motion produces a much more focused ensemble than an indirect constraint like the radius of gyration.

It becomes evident that a Rg bias can confine the search space but is certainly not sufficient to make a precise prediction of a distinct conformational state of a protein. Despite the relatively low tolerance of the Rg that we employed to generate the ensembles used for this study we find that they span a surprisingly large conformational space. However, we found that MD simulations are well-suited to distinguish between close-to-native conformations and decoys. Energetically unfavourable conformations are usually quickly forced to change their conformation and also their radius of gyration. Hence, they serve as reliable filter to determine conformations close to the native ligand bound state.
